# Adolescent perceptions of dissuasive sticks: a web survey among 16–20 year olds in Norway

**DOI:** 10.1186/s12889-018-5847-1

**Published:** 2018-08-06

**Authors:** Ingeborg Lund, Janne Scheffels

**Affiliations:** Norwegian Institute of Public Health, Department of Alcohol, Tobacco and Drugs, PO Box 4404, Nydalen, N-0403 Oslo, Norway

**Keywords:** Dissuasive cigarette sticks, Adolescents, Perceptions

## Abstract

**Background:**

While increasingly stringent rules for cigarette pack design restrict the advertising potential of cigarette packs, the cigarette stick itself remains a potential medium for marketing. Common design features are filters, slim cigarettes and capsule cigarettes. Recent research indicates lower general appeal, more negative perceptions of taste, and greater harm for cigarettes designed to be unappealing (dissuasive sticks), and the aim for the current study was to investigate perceptions of dissuasive cigarette sticks among Norwegian adolescents, and learn about factors that might make cigarettes unappealing to them.

**Methods:**

Two hundred eighty-one adolescents, 16–20 years old, smokers and non-smokers, assessed the appeal, taste, harmfulness, and which one they would most likely want to try, of 6 different cigarette sticks in a web survey. The cigarette sticks included two standard designs: cork and white filter sticks, and 4 dissuasive designs: green sticks, yellow sticks, and two white sticks with a health warning printed on the side.

**Results:**

All dissuasive designs were perceived as less appealing, worse tasting, more harmful than the standard cork tip and white tip cigarettes. The dissuasive sticks were less often chosen as a cigarette one would want to try. The evaluations of designs were relatively similar across gender, smoking and snus use status, and smoking susceptibility. In multinomial regressions, perceptions of taste and harm differences were associated with perceived product trial.

**Conclusions:**

This study supports earlier findings, and suggest that the use of unpleasant colours and warnings printed directly on cigarette sticks could increase perceived harmfulness, reduce notions of good taste, and possibly reduce desires to experiment with cigarettes in adolescence.

## Background

Today, common design features for cigarette sticks internationally include filters, filter colours, stick length and width, and the use of flavours or flavour capsules. The use of logos, colours and decorative elements printed directly on cigarette sticks has also been documented [1]. An influence on connotations of smoking from these types of design features is suggested in studies showing effects on perceptions of harmfulness and taste in both adults [2–4] and adolescents [5, 6]. White-tipped cigarettes were found to be perceived as less harmful in O’Connor et al. [3], slim and super-slim cigarettes tended to be more popular among adolescents in Ford et al. [6], while a preference for capsule cigarettes has been demonstrated for adolescents [5, 7] and adult smokers [4].

In order to reduce the marketing potential of cigarette sticks, researchers have begun to explore consumer perceptions of cigarettes designed to be dissuasive. With the help of a distasteful, unpleasant looking, cigarette – the so-called dissuasive stick – it is hypothesised that negative connotations of smoking will increase, and that this potentially might reduce interest in smoking in the population [8]. In recent research, assessments of appeal and harmfulness of dissuasive cigarette sticks have been explored in experimental settings in New Zealand and the UK. In New Zealand, Hoek et al. [8] found that dissuasive sticks were viewed more negatively by adult smokers, while in a qualitative study among young adult female smokers, Hoek and Robertson [9] found that dissuasive sticks challenged connotations of cleanliness, and connoted stereotypes that participants wanted to avoid. Specifically for adolescents, results from the UK showed support for a cigarette with a ‘smoking kills’ warning label printed on it, as it was believed that it would deter smoking [10], while two dissuasive designs (smoking kills and green colour) was rated less favourably than the standard cigarette by UK young adults [11].

The aim of the current study was to investigate perceptions of dissuasive and standard cigarette sticks among Norwegian adolescents, and learn about factors that might make cigarettes unappealing to them. Based on previous results it is hypothesised that dissuasive sticks will be less appealing, be understood as more harmful, and less often be chosen as a cigarette to try by adolescents. As smoking starts with experimenting, a particular focus will be on what factors that might influence adolescents’ interest in trying a cigarette stick.

## Methods

The data collection was carried out by the commercial pollster Epinion during 2016, drawing respondents from their existing web panel of 80,000 individuals. To reduce risks of self-selection, participation in the panel is by invitation only and, as a reward for survey participation, respondents get to partake in a raffle for gift vouchers. Epinion adheres to the ICC/ESOMAR international code on market and social research, which includes guidelines for data collection, data management, and ethical procedures. The project was approved by the Data Protection Officer at the Norwegian Institute of Public Health. Individuals were non-identifiable in the data set, and no sensitive information was collected. An introductory statement made it clear that participation was voluntary, and explained the purpose of the survey.

This was a cross-sectional survey, and participation entailed filling out a web-questionnaire. An automatic sampling procedure, sending invitations on an ongoing basis and according to the degree of completion of demographic segments, as compared to official population statistics, enabled purposeful sampling in groups with a lower degree of participation during the data collection phase.

From a total sample of 919 individuals between 16 and 75 years old, all respondents younger than 21 years were selected for the current analyses. The analytic sample (*N* = 281) includes both smokers and non-smokers. Due to large difficulties in recruiting boys, the sample includes an overweight of females (Table 1), and representativity can therefore not be ascertained.

**Table 1:** Descriptives (*N* = 280)

		N	%
Gender	Female	197	70.4
	Male	83	29.6
Age group	16–18	132	47.1
	18–21	148	52.9
Smoking susceptibility^a^	no	147	61.0
	yes	94	39.0
Current smoking	no	240	85.7
	yes	40	14.3
Current snus use	no	213	76.1
	yes	67	23.9

^a^Smokers (*n* = 40) are not included

**Fig. 1:**
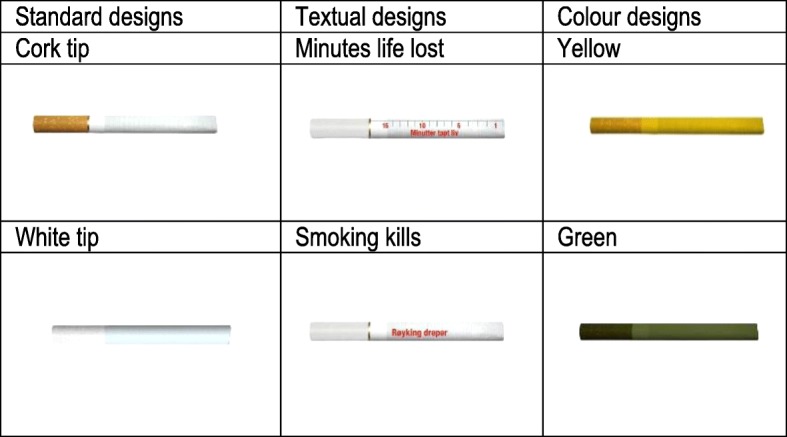
Cigarette stick designs* used in the survey

All respondents were first shown images of 6 cigarette sticks with different designs (Fig. 1) in random order for individual assessment. Then all 6 sticks were displayed together for the purpose of three comparison tasks. The design of the cigarettes was adapted from Hoek et al. [8] for a Norwegian sample by translation of the textual warning labels into Norwegian. None of the cigarettes displayed a brand name.

*Images created by the authors for this study. Only the two standard designs are commercially available in the Norwegian market.

### Variables

#### Outcome variables

##### Appeal

Cigarette sticks were rated individually according to their general appeal, with answer categories ranging from 1 – very low appeal, to 11 – very high appeal. For the regression analysis, due to low statistical power, two dummy variables were constructed based on appeal scores below (0) or on or above (1) the median score for 1) dissuasive designs and 2) standard designs.

##### Taste, harm and trial

In three comparison tasks, all six sticks were presented simultaneously. Respondents were asked to nominate the best tasting cigarette, the most harmful cigarette, and the cigarette they would most likely want to try. All these comparison tasks included the option ‘no difference between designs’. For regression analysis, three categorical variables were constructed. In our interpretation, an expressed wish to try one of the dissuasive cigarettes implies that the individual is not dissuaded by these designs. Furthermore, based on research showing that brand and marketing awareness [12] and brand identity [13] are linked to risk of smoking, the wish to try a standard cigarette was construed as a sign of higher smoking interest than the no preference option, which was understood as less interest. Consequently, three categories were constructed, separating those who wanted to try one of the dissuasive designs, i.e. the non-dissuaded individuals (0), from those who had no preference (1) or who preferred a standard design (2). For harmfulness, three categories denote the perceptions ‘there is no harm difference between designs’ (0), ‘a standard design is the most harmful’ (1) or, ‘a dissuasive design is the most harmful’ (2). For taste, three categories reflect the responses ‘there is no taste difference between designs’ (0), ‘a standard design has the best taste’ (1) or, ‘a dissuasive design has the best taste’ (2).

#### Control variables

*Current smoking* was measured by the question ‘Do you smoke’, with answer categories ‘yes daily’, ‘yes occasionally’, and ‘no’. Due to small group sizes, all daily and occasional smokers were categorised as current smokers in the analyses.

##### Smoking susceptibility

Three questions were included to measure susceptibility to smoking among non-smokers (would you smoke a cigarette if offered to you by a friend, do you think you will smoke a cigarette during the next 12 months, do you think you will smoke cigarettes when you are older). Answer categories were ‘definitely not’, ‘probably not’, ‘uncertain’, ‘probably’, and ‘definitely’. All respondents who gave another answer than ‘definitely not’ to any of these questions were categorised as susceptible to smoking.

For regression analyses, smoking status and smoking susceptibility were joined together, constructing the three point *smoking status* scale: non-susceptible non-smoker (0), susceptible non-smoker (1), and current smoker (2).

*Current snus (Swedish moist snuff) use* was measured similarly to smoking, and all daily and occasional snus users were categorised as current snus users.

##### Demographics

The respondents’ gender and age were recorded.

### Analyses

Mean appeal scores were compared between cigarette designs using paired samples t-tests. Analysis of variance (ANOVA) was applied to look for differences in group means. Chi-square testing was used to find significant differences in the proportions preferring each design along the dimensions taste, wish to try, and harmfulness. An adjusted multinomial regression, entering all independent variables simultaneously, was applied to find correlates to the wish to try a dissuasive design (non-dissuaded), and the wish to try a standard design, with the reference group being to have no try-preference. Independent variables were age, gender, smoking status, current snus use, categorical appeal of dissuasive and standard sticks, and perceived harm and taste differences.

## Results

The analytic sample included 281 adolescents, 16–20 years old. Just over 70% of the participants were female, and almost half were younger than 18 years (Table 1). Altogether 39% of the non-smokers (amounting to 34% of the entire sample) were defined as susceptible to smoking. The proportion of daily and occasional smokers (14%) was lower than the proportion of daily and occasional snus users (24%).

In individual stick assessments (Table 2), the average appeal scores obtained by the cork tip and the white tip cigarette designs were significantly higher than the scores obtained by any other design (*p* < .001). There was no significant difference in appeal scores between cork and white tips, between the two textual designs, or between the yellow and green designs. However, the dissuasive colour sticks received a significantly lower appeal score than the dissuasive textual sticks (*p* < .001).

**Table 2:** Average appeal score overall and in segments (scale: 0–11)

	Cork tip	White tip	Min. life lost	Smoking kills	Yellow	Green
Overall mean appeal score^a^	4.54	4.74	3.58	3.78	2.61	2.39
Mean appeal score in segments						
Gender						
Female	4.59	4.94	3.34	3.69	2.46	2.40
Male	4.37	4.31	4.15	3.95	3.01	2.37
Age groups						
16–18	4.49	4.73	4.10*	4.07	2.80	2.53
18–21	4.58	4.78	3.12	3.52	2.45	2.28
Smoking susceptibility						
Yes	5.44***	5.35***	3.90	4.17**	2.87**	2.57*
No	3.18	3.64	3.22	3.10	2.05	1.90
Current smoking						
Yes	7.40***	7.45***	4.13	5.33***	4.10***	3.82***
No	4.06	4.31	3.49	3.52	2.37	2.16
Current snus use						
Yes	5.85***	6.28***	3.89	4.45*	2.77**	3.03
No	4.13	4.28	3.48	3.57	2.57	2.20

^a^t-test (paired samples): *p* < .001 between standard and all dissuasive designs, *p* < .001 between white and all dissuasive designs, *p* < .001 between yellow and textual dissuasive, *p* < .001 between green and textual dissuasives, *p* < .001 between min. Life lost and colour dissuasives. No sign. Difference between standard and white, between yellow and green, or between min. Life lost and smoking kills

*: ANOVA, *p* < 0.05. **: ANOVA, *p* < 0.01. ***: ANOVA, *p* < 0.001

With the exception of the minutes life lost-design, current smokers found all designs significantly more appealing than current non-smokers, and susceptible non-smokers found them significantly more appealing than non-susceptible non-smokers. With the exception of the minutes life lost and the green designs, current snus users found all designs significantly more appealing than current non-snus-users (Table 2). Age was a significant factor only for the minutes life lost design, which was rated significantly more appealing by 16–18 year olds than by 18–21 year olds. There were no significant gender differences.

The ordering of sticks was consistent across sub-groups, and standard designs achieved the highest score in all segments, with the lowest scores given by non-susceptible non-smokers (white: 3.6, cork: 3.2), and the highest by current smokers (white: 7.5, cork: 7.4). The yellow and green cigarettes were deemed to be the least appealing in all segments, with the lowest scores given by non-susceptible non-smokers (yellow: 2.05, green: 1.90), and the highest by current smokers (yellow: 4.1, green: 3.8). The appeal scores given to cigarettes with warning texts fell between standard and colour design-scores, and ranged from 3.1 (non-susceptible non-smokers) to 5.3 (current smokers) for the smoking kills cigarette, and from 3.3 (female) to 4.2 (male) for the minutes life lost cigarette.

In a direct comparison of all 6 designs (Table 3), most respondents believed there was no taste difference (36% overall), they believed the white cigarette had the best taste (34% overall), or they believed the cork cigarette had the best taste (25% overall). Very few respondents thought the best tasting cigarette was to be found among any of the dissuasive designs, with the yellow stick being the most frequent dissuasive choice, nominated by 2% of the sample. Although there were differences in the preferences of different segments, the only significant group difference was found between smokers and non-smokers (*p* < .01). Smokers more often preferred the cork tip (32% vs 24%) or the yellow cigarette (8% vs 1%), and more seldom thought there was no taste difference between designs (24% vs 37%).

**Table 3:** Results from direct comparison tasks ^a^

	Standard	White	Min. life lost	Smoking kills	Yellow	Green	No difference
Which cigarette has the best taste?	%	%	%	%	%	%	%
Total sample (*N* = 241)	25.3	33.6	1.2	0.4	2.1	1.7	35.7
Gender							
Female	22.5	38.5	0.6	0.6	1.8	2.4	33.7
Male	31.0	22.5	2.8	0.0	2.8	0.0	40.8
Susceptible to smoking^b^							
No	17.2	36.1	0.8	0.0	0.8	1.6	43.4
Yes	34.1	31.7	1.2	0.0	1.2	2.4	29.3
Current smoking							
No	24.0	34.3	1.0	0.0	1.0	2.0	37.7
Yes**	32.4	29.7	2.7	2.7	8.1	0.0	24.3
Current snus use							
No	24.4	31.7	1.1	0.0	2.8	1.7	38.3
Yes	27.9	39.3	1.6	1.6	0.0	1.6	27.9
Which cigarette would you want to try?							
Total sample (*N* = 249)	22.5	43.0	6.8	0.8	2.0	5.6	19.3
Gender							
Female	20.6	50.9	4.6	0.6	2.3	2.9	18.3
Male***	27.4	23.3	12.3	1.4	1.4	12.3	21.9
Susceptible to smoking^b^							
No	17.2	39.1	7.0	0.8	2.3	2.3	31.3
Yes**	33.7	43.4	4.8	1.2	1.2	7.2	8.4
Current smoking							
No	23.7	40.8	6.2	0.9	1.9	4.3	22.3
Yes*	15.8	55.3	10.5	0.0	2.6	13.2	2.6
Current snus use							
No	25.7	40.6	6.4	0.5	2.7	3.2	20.9
Yes*	12.9	50.0	8.1	1.6	0.0	12.9	14.5
Which cigarette looks more harmful?							
Total sample (*N* = 277)	2.5	0.4	18.4	1.8	22.4	43.3	11.2
Gender							
Female	1.6	0.5	19.2	1.0	23.8	43.0	10.9
Male	4.8	0.0	16.9	3.6	18.1	44.6	12.0
Susceptible to smoking^b^							
No	3.5	0.0	16.1	2.1	17.5	44.8	16.1
Yes	1.1	0.0	20.2	2.1	24.5	45.7	6.4
Current smoking							
No	2.5	0.0	17.7	2.1	20.3	45.1	12.2
Yes*	2.5	2.5	22.5	0.0	35.0	32.5	5.0
Current snus use							
No	2.4	0.5	19.0	1.9	20.5	44.3	11.4
Yes	3.0	0.0	16.4	1.5	28.4	40.3	10.4

Chi square group differences: *: *p* < .05; **: *p* < .01; ***: *p* < .001. ^a^ Each section shows results from 4 independent analyses

^b^Smokers are not included in the susceptible to smoking analysis

Overall, 44% of the sample perceived the green design as the most harmful cigarette, while 22% nominated the yellow design, and 19% the minutes life lost design (Table 3). The white tip (0.4%) and the smoking kills cigarettes (2%) were least likely to be perceived as the most harmful choices. The proportions maintaining that there was no harm difference between designs were relatively small, and significantly smaller among smokers (5%) than among non-smokers (12%; *p* < .05). Smokers were also significantly less likely to think that the green cigarette would be the most harmful, compared to non-smokers (33% vs 45%; *p* < .05).

The white tip cigarette was the most common design to want to try, both overall (42%), and in most sub-groups (Table 3). The highest proportion to nominate this design was found among current smokers (55%), followed by females (51%) and current snus users (50%). The lowest proportion was among males (23%).

Although the most common choice of product to try was to prefer a standard cigarette (58% of sample, Table 4), or to have no preference (28% of sample), there were significant differences between all segments on this question (*p* < .05 or better). In some groups, the proportions nominating dissuasive designs did reach a certain magnitude. This was particularly the case for males nominating the green (12%) or the minutes life lost (13%) designs, current smokers nominating the green (13%) or the minutes life lost designs (11%), and current snus users nominating the green design (13%). Moreover, for smokers and snus users, the percentages nominating a dissuasive design were almost equal to the percentages nominating a cork tip design to try (for cork tip: smokers, 16%; snus users, 13%).

**Table 4:** Correlates of preferring to try a standard or a dissuasive design (ref = no preference) *N* = 278

	Prefer standard vs no preference (58.3%)			Prefer dissuasive vs no preference (13.7%)		
	AOR	95% CI for AOR		AOR	95% CI for AOR	
Intercept						
Age	1.06	0.81	−1.39	1.14	0.78	−1.67
Female	1.93	0.87	−4.27	0.37	0.13	−1.03
Low dissuasive appeal	0.71	0.31	−1.62	0.46	0.14	−1.45
Low standard appeal	0.32**	0.13	−0.79	0.40	0.12	−1.34
Taste difference (ref = no difference between designs)						
Dissuasive best taste	4.11	0.29	−59.23	54.10***	3.77	− 775.59
Standard best taste	9.01***	4.08	−19.94	3.34*	1.14	−9.83
Harm difference (ref = no difference between designs)						
Dissuasive most harmful	5.81***	1.97	−17.10	16.71**	1.76	− 158.70
Standard most harmful	3.60	0.21	−62.61	129.49***	4.54	− 3690.86
Smoking status (ref = non-susceptible non-smoker)						
Smoker	3.54	0.72	−17.39	2.38	0.36	−15.65
Susceptible	2.21	0.96	−5.08	1.25	0.38	−4.08
Snus user	0.61	0.22	−1.67	2.19	0.62	−7.74

All independent variables entered simultaneously

*AOR* Adjusted Odds Ratio, *CI* Confidence interval, *: *p* < .05; **: *p* < .01; ***: *p* < .001

Even though no smoker believed that the smoking kills cigarette would be the most harmful, no smoker wanted to try that particular design.

Results from a multinomial regression (Table 4) showed that perceived low appeal of standard designs lowered the likelihood for wanting to try a standard design versus not having a try-preference (AOR = .32, *p* < .01). Thinking that one of the standard designs had the best taste (AOR = 9.01, *p* < .001), or that a dissuasive stick was the most harmful cigarette (AOR =5.81, *p* < .001) increased the likelihood of nominating a standard stick to try. Gender, age, smoking or snus use status was not associated with choosing a standard stick to try versus not having a preference.

Respondents who believed there would be a taste difference between designs were more likely to want to try a dissuasive stick compared to those who thought that there was no taste difference. While the largest effect was seen for perceiving that a dissuasive stick had the best taste (AOR = 54.10, *p* < .001), there was also a positive effect of perceiving that a standard stick had the best taste (AOR = 3.34, *p* < .05). Similarly, a perception of harm differences between designs was associated with increased likelihood of wanting to try a dissuasive stick, compared to not perceiving any harm difference between designs. The largest effect was seen for those who thought a standard stick was the most harmful (129.49, *p* < .001), but there was also a strong effect for those who thought a dissuasive stick was the most harmful (AOR = 16.71, *p* < .01). Gender age, product appeal, smoking and snus use status were not statistically associated with the wish to try a dissuasive stick.

## Discussion

In this study, Norwegian adolescents rated cigarette sticks designed to look aversive as less appealing and more harmful than standard cork tip and white tip cigarettes. When prompted to choose a stick to potentially try, less than 14% of them picked a dissuasive stick. Factors associated with an increased likelihood of dissuasive product trial were perceptions of harm and taste differences between designs.

There was statistically non-significant variability in the evaluations of individual dissuasive designs, with dissuasive colour sticks tending to obtain a somewhat lower appeal score and higher harm-evaluation, than white sticks with dissuasive text messages printed on them. For product trial, the situation was slightly different, with the green colour design and the minutes life lost designs being nominated by larger proportions than the yellow design and the smoking kills designs.

A tendency for dissuasive sticks to be rated as less appealing than traditional sticks is a finding supported by previous research [8–11]. However, despite an overall agreement in results between this and earlier studies, there are some differences in the details. In particular, while New Zealand adult smokers found the minutes life lost cigarette to be the most unappealing of the dissuasive designs [8], our Norwegian adolescents rated the green and yellow sticks lowest on appeal. This might possibly reflect the differences in age segments and smoking status between the two studies. One might speculate that the assessments of our group of young, mostly non-smokers, would be more influenced by challenges to ideas of cleanliness [9] than the adult smokers in the New Zealand study, as the latter group were accustomed to smoking and perhaps less concerned with factors like bad smell.

Several of our results indicate that the interest to try a cigarette is influenced by other factors than just perceptions of taste, harm and appeal. For example, males, smokers and snus users were as likely as others to think that the minutes lost cigarette and the green cigarette were harmful, but their interest in trying them were still higher than in other groups. Correspondingly, while smokers thought the smoking kills design was more appealing than other dissuasive designs, and no smoker thought it was the most harmful cigarette, none of them was interested in trying it. A possible explanation for this last effect, suggested by earlier findings, is that although the design is not viewed as unappealing as such, using it in public is, as it would ruin the image of smoking for young people [14]. Results by Moodie et al. [15] also suggest that female young smokers might be deterred by the perceived social discomfort of being seen smoking such a cigarette. Both these examples underline the fact that while perceptions of taste, harm and appeal are intra personal ideas, using a cigarette is inter personal. Smoking has a social dimension to it, which might moderate any effects on interest in particular design from the individual assessments of taste and harm.

Still, after controlling for other factors, adolescents who thought dissuasive sticks looked less harmful and better tasting were more likely to express an interest in trying them. However, even respondents who perceived dissuasive sticks as more harmful and not best in taste were less dissuaded by them than individuals who thought all sticks were equally harmful and had equal taste. Perceiving cigarettes as equal in terms of harm and taste was in other words more closely associated with a disinterest in product trial than any perception of variation. Differentiation between cigarettes in itself might thus be a factor that could inspire an interest in product trial, possibly due to a “curiosity-to-try” factor, which has been found also for flavoured cigarettes [16].

### Limitations

This is a new field of research, and results need validation from other studies for increased reliability. For this study, an overrepresentation of females reduces generalisability to the adolescent population. Moreover, small group sizes were an important factor behind the large confidence intervals for dissuasive taste and standard harmfulness in the regression analysis. It also made it necessary to combine daily and occasional smokers into one group. However, given the very low prevalence of smoking in this age segment, it is challenging to recruit larger groups of smokers. The use of e-cigarettes was very low among adolescents at the time of the data collection and was not measured. However, future research should also include this information.

When interpreting the result, one should keep in mind that the respondents had not seen any of the dissuasive designs before. This may have made for increased curiosity, and potentially increased the tendency to nominate dissuasive sticks to try, or resulted in higher dissuasive stick appeal scores. In addition, even though there was a “no preference” option, some respondents may have felt prompted by the situation to choose a design. On the other hand, as this was self-reported data, social desirability bias might have resulted in lower appeal scores, or lowered expressions of interest in trying any type of cigarette.

## Conclusion

This study indicates that unpleasant colours and warning labels printed directly on cigarette sticks could increase perceived harmfulness, make adolescents think that the product has a worse taste, and possibly reduce desire to experiment with cigarettes. Adolescence is the period when smoking uptake usually happens. Consequently, to reduce the appeal of, and the interest in trying, cigarettes in this age group could potentially lead to less interest in taking up smoking and increased interest in quitting.

## Abbreviations

AOR: Adjusted Odds Ratios
CI: Confidence Interval
ICC: International Chamber of Commerce
